# Clinical profiles of subclinical disease among pulmonary tuberculosis patients: a prospective cohort study in South Korea

**DOI:** 10.1186/s12890-020-01351-z

**Published:** 2020-12-02

**Authors:** Jinsoo Min, Chaeuk Chung, Sung Soo Jung, Hye Kyeong Park, Sung-Soon Lee, Ki Man Lee

**Affiliations:** 1 Division of Pulmonary and Critical Care Medicine, Department of Internal Medicine, Daejeon St. Mary’s Hospital, College of Medicine, The Catholic University of Korea, Seoul, Republic of Korea; 2 Division of Pulmonary and Critical Care Medicine, Department of Internal Medicine, Chungnam National University Hospital, Daejeon, Republic of Korea; 3 Division of Pulmonary and Critical Care Medicine, Department of Internal Medicine, Ilsan Paik Hospital, Inje University College of Medicine, Goyang, Republic of Korea; 4 Department of Internal Medicine, Chungbuk National University College of Medicine, 1, Chungdae-ro, Seowon-gu, Cheongju, 28644 Republic of Korea; 5 Division of Pulmonary and Critical Care Medicine, Department of Internal Medicine, Chungbuk National University Hospital, Cheongju, Republic of Korea

**Keywords:** Pulmonary tuberculosis, Symptom, Computed tomography, Bronchoscopy, Subclinical disease

## Abstract

**Background:**

Subclinical tuberculosis (TB) is a potential target for public health intervention because its early identification may reduce TB transmission. We aimed to describe the clinical and laboratory findings of subclinical disease among pulmonary TB patients and compared treatment outcomes for subclinical and active diseases.

**Methods:**

In this prospective cohort study, we enrolled adult patients aged ≥ 19 years with pulmonary TB between 2016 and 2018. Subclinical TB was defined as radiographic or microbiologic test results consistent with TB without clinical symptoms. We implemented a two-stage symptom assessment using a predefined TB symptom checklist. Demographic, clinical, and laboratory data were compared between subclinical and active diseases using multivariable binary logistic regression analysis. We evaluated treatment outcomes in the drug-susceptible cohort.

**Results:**

Among 420 enrolled patients, 81 (19.3%) had subclinical TB. Multivariable analysis showed that age < 65 years was the only variable significantly associated with subclinical disease. Subclinical disease had a significantly lower proportion of acid-fast bacilli smear and culture positivity and multiple lobe involvement compared to active disease. The white blood cell counts, platelet counts, and C-reactive protein levels were significantly higher among patients with active disease than among those with subclinical disease. Among 319 patients with treatment success in the drug-susceptible cohort, six (1.9%) recurrent cases were identified, and all were active disease. Patients with subclinical disease had a higher proportion of favourable outcomes; however, its odds ratio was insignificant.

**Conclusions:**

Nearly one-fifth of tuberculosis cases were subclinical in South Korea. Despite its milder clinical presentation and lower level of inflammatory markers, the treatment outcomes of subclinical TB were not significantly different from that of active disease.

## Background

It is estimated that one-fourth of the world’s population is infected with *Mycobacterium tuberculosis* [1]. The ‘End TB Strategy’ of the World Health Organization (WHO) seeks to reduce tuberculosis (TB) incidence by 90% and TB deaths by 95% by 2035 [2]. The key approaches are optimum use of existing interventions, availability, and wide use of new tools to improve efforts to find and treat people with active TB, and universal screening of individuals at high risk. Thus, the diagnosis of subclinical TB, which could allow the treatment of individuals before they become symptomatic and infectious, has been highlighted as essential to make significant progress for the WHO’s target.

Recent research has demonstrated that human TB infection exists within a continuous spectrum of bacterial metabolic activities and antagonistic immunological responses from latent TB infection to active TB disease [3]. Latent TB infection, which undergoes an imbalance of bacterial activities and host defences, leads to disease progression through a subclinical phase [4]. Subclinical TB disease is due to viable *Mycobacterial tuberculosis* infection that does not cause clinical TB-related symptoms but causes other abnormalities that can be detected using existing radiologic or microbiologic assays [3].

In South Korea, with the highest TB incidence among the high-income countries [5], TB screening using chest radiography is regularly performed for adults as part of health examinations for health insurance subscribers [6]. It is mandatory for new employees of healthcare institutions, schools, nursery, and social welfare facilities to undergo TB screening during pre-employment medical check-up [7]. In addition, the government of South Korea recently strengthened the strategies of TB elimination, which highlighted the early detection of TB infection in vulnerable populations such as older and homeless people [8]. These health policies in South Korea have increased subclinical TB detection; however, its clinical characteristics and outcomes are not well understood. We hypothesized that subclinical TB would have a milder disease course with a lower bacterial burden and better clinical outcomes than active TB. Thus, we aimed to describe the clinical and laboratory findings of subclinical disease among pulmonary TB patients and compared treatment outcomes for subclinical and active diseases.

## Methods

### Study design and participants

We enrolled adult patients with pulmonary TB from the cohort study of pulmonary tuberculosis (COSMOTB) between November 2016 and September 2018 to compare the clinical characteristics of active and subclinical TB. Briefly, COSMOTB is a prospective observational cohort study to assess the prevalence of discordant results of phenotypic and molecular drug susceptibility tests [9]. COSMOTB was conducted at three university-affiliated tertiary hospitals in South Korea that participated in the public–private mix project for TB control in South Korea. TB specialist nurses under this project educated TB patients and monitored them for medication adherence and adverse drug reactions. The inclusion criteria are as follows: (1) age ≥ 19 years, (2) a diagnosis or suspicion of pulmonary TB, and (3) receiving anti-TB treatment for less than one month. The exclusion criteria are as follows: (1) age ≤ 18 years, (2) extrapulmonary TB without pulmonary involvement, (3) patients who were finally diagnosed as inactive TB or pulmonary diseases other than TB, and (4) voluntary withdrawal from study participation. Inactive TB was diagnosed when a follow-up chest radiography showed no pulmonary lesions changes or if previous chest images revealed unchanged lesions without microbiological evidence of *M. tuberculosis* infection [10]. We used a convenience sampling method to approach and recruit study participants.

### Definition of subclinical and active diseases

Patients were categorized as having active TB or subclinical TB. Active TB was defined as the presence of clinical TB-related symptoms with radiographic abnormalities or microbiologic evidence of *M. tuberculosis*. Subclinical TB was defined as the presence of radiographic or microbiologic test results consistent with TB without clinical symptoms. We implemented a two-stage symptom assessment using a predefined checklist, which listed TB-related symptoms, such as cough, sputum, fever, general weakness, dyspnoea, chest pain, body weight loss, and haemoptysis. First, TB patients met a TB specialist nurse at the hospital, who interviewed and identified patients’ TB-related symptoms. Subsequently, patients met with a physician at the clinic, who reconfirmed their symptoms and their duration. As patients were identified as asymptomatic after two-stage assessment, they were categorized as subclinical TB disease.

### Data collection

Participants were evaluated at each hospital on study entry. Demographic, clinical, and laboratory data were prospectively collected from enrolled patients using a case report form upon study entry. Microbiological tests were performed after the first clinical assessment by a physician. Acid-fast bacilli (AFB) smears using light and fluorescent microscopy and nucleic acid amplification test (NAAT) were conducted at each hospital. *Mycobacterium* culture testing using both solid (3% Ogawa media) and liquid (BACTEC MGIT 960 system, BD, NJ, USA) cultures were performed at the reference laboratory. Culture-based phenotypic drug susceptibility tests were performed using the absolute concentration method on Löwenstein–Jensen medium.

### Statistical analyses

Continuous variables were presented as means and standard deviations or medians and interquartile ranges, whereas discrete variables were presented as frequencies or percentages. The baseline characteristics of patients with active or subclinical TB were compared; univariable analysis was performed using Chi-square test for categorical variables and Mann–Whitney U test for continuous variables. We calculated the lower and upper limits of the 95% confidence intervals for a proportion using the VassarStats (a website for statistical computation), wherein the Wilson procedure without a correction for continuity was used. Subsequently, we selected age, sex, and other clinical variables with *p* values < 0.20 [11] based on the univariable analysis and further performed multivariable binary logistic regression to evaluate the possible association between variables and subclinical TB. For regression, we used a complete-case analysis approach and unknown data were regarded as missing values. A *p* value < 0.05 was considered statistically significant. All statistical analyses were performed using SPSS version 17.0 (Statistical Product and Service Solutions, Chicago, IL, USA).

#### Sample size

We selected eight variables a priori for inclusion into our model, such as age at diagnosis, sex, foreigners, body mass index, chronic respiratory disease, AFB smear, culture, and NAAT results. Eighty events of subclinical diseases are required to ensure a minimum of 10 events per variable, which are needed to minimize bias in logistic regression models [12]. Assuming that proportions of subclinical disease are 18–21% [3], 381–445 patients with pulmonary TB were required for sample size.

#### Treatment outcomes

Participants were evaluated at 2 and 4 weeks, 2, 4, 6, 9, 12, and 24 months after initiating anti-TB treatment to document their treatment outcome. Those with successful outcomes were also followed for at least 1 year to identify recurrence. When patients could not visit the clinic during the study period, we contacted them on the phone. If patients complained of any TB-related symptoms during the post-treatment follow-up period, we advised them to visit the clinic as soon as possible. If we could not reach the patients after transfer-out, we contacted the healthcare staffs of the hospital where TB patients had been transferred to. Treatment outcomes were defined according to the Korean TB guidelines adopted from the WHO’s definition [13]. Treatment success was the sum of cured patients and those that completed treatment within 1 year of anti-TB treatment. Favourable outcome was defined as patients who had achieved treatment success without recurrence within the 1-year post-treatment follow-up period. We evaluated the association between subclinical disease and treatment outcome in the drug-susceptible cohort comprised of patients with positive culture results susceptible to both isoniazid and rifampin and clinically diagnosed TB patients without microbiological evidence using binary logistic regression and adjusting for age and sex.

## Results

**Fig. 1:**
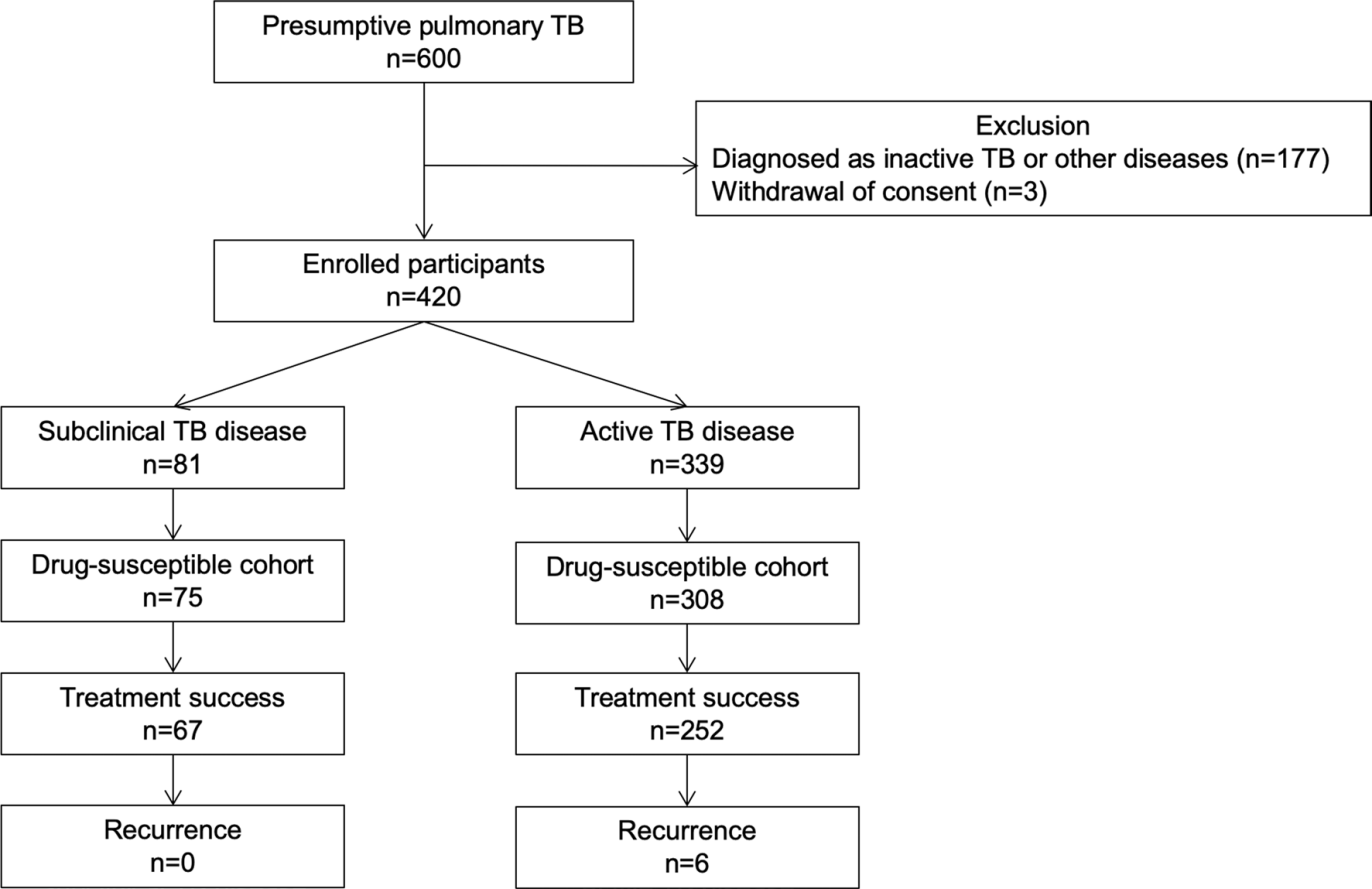
Flow chart of patient enrollment and final outcomes of drug-susceptible cohort. *TB* tuberculosis. ^1^Drug-susceptible cohort comprises patients who have positive culture results susceptible with both isoniazid and rifampin and who are clinically diagnosed with tuberculosis without microbiological evidence. ^2^Inactive TB was diagnosed when a follow-up chest radiography showed no changes of pulmonary lesions or a previous chest images revealed unchanged lesions without microbiological evidence of *Mycobacterium tuberculosis* infection

After screening 600 patients with presumptive pulmonary TB, 339 patients with active disease and 81 patients with subclinical disease were finally enrolled in this study (Fig. 1). Table 1 summarizes the baseline characteristics of the 420 enrolled patients. The mean age was 59.2 ± 19.6 years, and 258 (61.4%) were men. Patients with subclinical TB were younger than those with active TB (51.9 ± 19.2 vs. 61.0 ± 19.3 years, *p* = 0.000). The prevalence of chronic pulmonary disease (8.3% vs. 2.5%, *p* = 0.069) and prior TB history (17.7% vs. 18.5%, *p* = 0.863) was similar between patients with active and subclinical diseases. The positivity of AFB smear (31.0% vs. 13.6%, *p* = 0.002) and culture tests (72.3% vs. 46.2%, *p* = 0.002) and NAAT (70.1% vs. 46.2%, *p* = 0.000) among patients with active disease was significantly higher than that among patients with subclinical disease (Table 2). The white blood cell counts, platelet counts, and C-reactive protein levels were significantly higher among patients with active disease than among those with subclinical disease. The haemoglobin level was significantly lower among male patients with active disease than among male patients with subclinical disease. Multivariable analysis showed that age < 65 years was the only significant variable associated with subclinical disease, and the positivity of initial NAAT was significantly associated with active disease (Table 3).

**Table 1:** Baseline characteristics of enrolled patients with active and subclinical TB disease

Variables	All patients (n = 420)	Active TB disease (n = 339)	Subclinical TB disease (n = 81)
Male	258 (56.7–66.0%)	208 (56.1–66.4%)	50 (50.8–71.6%)
Age ≥ 65 years	183 (38.9–48.4%)	161 (42.2–52.8%)	22 (18.7–37.7%)
Foreigner	17 (2.5–6.4%)	10 (1.6–5.3%)	7 (4.2–16.8%)
Body mass index (kg/m^2^)^a^			
< 18.5	65 (12.5–19.6%)	58 (13.7–21.8%)	7 (4.4–17.2%)
≥ 18.5	348 (80.4–87.5%)	276 (78.2–86.3%)	72 (82.8–95.6%)
Comorbidities			
Chronic respiratory disease	30 (5.0–10.0%)	28 (5.8–11.7%)	2 (0.7–8.6%)
Diabetes mellitus	75 (14.5–21.8%)	60 (14.0–22.1%)	15 (11.6–28.3%)
Prior TB history	83 (16.2–23.8%)	70 (16.7–25.3%)	13 (9.6–25.5%)

Values were expressed as numbers with 95% confidence intervals of their proportion

*TB* tuberculosis

^a^Unknown data are regarded as missing

**Table 2:** Laboratory and microbiological findings of enrolled patients with active and subclinical TB disease

Variables	All patients (n = 420)	Active TB disease (n = 339)	Subclinical TB disease (n = 81)	*p* value
Initial AFB smear test result				
Positive	116 (23.6–32.1%)	105 (26.3–36.1%)	11 (15.2–7.5%)	
Negative	304 (67.9–76.4%)	234 (63.9–73.7%)	70 (77.3–92.2%)	
Initial AFB culture test result				
Positive	289 (64.2–73.1%)	245 (67.3–76.8%)	44 (43.5–64.7%)	
Negative	131 (26.9–35.8%)	94 (23.2–32.7%)	37 (35.3–56.5%)	
Initial NAAT result^a^				
Positive	264 (60.7–70.0%)	228 (65.0–74.9%)	36 (35.5–57.1%)	
Negative	139 (30.0–39.3%)	97 (25.1–35.0%)	42 (42.9–64.5%)	
Drug susceptible test^a^				
Susceptible to both INH and RIF	249 (82.7–90.5%)	212 (82.5–90.9%)	37 (72.7–93.4%)	
Resistant to either INH or RIF	37(9.5–17.3%)	31 (9.1–17.5%)	6 (6.6–27.3%)	
Inflammatory markers^b^				
White blood cell count (mm^3^)	6970 ± 4084	7707 ± 3283	6137 ± 2626	0.000
Neutrophil (%)	58.6 ± 24.4	65.9 ± 16.2	58.2 ± 19.4	0.000
Lymphocyte (%)	22.5 ± 11.1	21.3 ± 11.2	26.7 ± 9.2	0.000
Platelet count (mm^3^)	248,368 ± 135,229	282,861 ± 122,293	225,926 ± 95,965	0.000
C-reactive protein (mg/dL)	2.7 ± 4.9	3.5 ± 5.2	0.5 ± 1.5	0.000
Haemoglobin (g/dL)^c^				
All participants	12.7 ± 1.9	12.6 ± 1.9	13.5 ± 1.9	0.000
Male	13.2 ± 2.1	13.1 ± 2.0	14.2 ± 1.8	0.001
Female	12.0 ± 1.5	11.9 ± 1.4	12.4 ± 1.3	0.087

Values were expressed as numbers with 95% confidence intervals of their proportion

*TB* tuberculosis, *AFB* acid-fast bacilli, *NAAT* nucleic acid amplification test, *INH* isoniazid, *RIF* rifampicin

^a^Unknown data are regarded as missing

^b^Mann–Whitney U test was conducted for white blood cell count, neutrophil, lymphocyte, platelet count, and C-reactive protein

^c^Student’s *t* test was conducted for haemoglobin

**Table 3:** Multivariable analysis for factors associated with subclinical tuberculosis diseases compared to active tuberculosis disease

Variables	Adjusted OR (95% CI)	*p* value
Male	1.11 (0.65–1.94)	0.690
Age < 65 years	2.12 (1.18–3.82)	0.012
Foreigners	2.40 (0.77–7.46)	0.129
BMI < 18.5 kg/m^2^	0.60 (0.25–1.45)	0.255
Chronic respiratory diseases	0.36 (0.08–1.60)	0.180
Initial AFB smear test (+)	0.56 (0.25–1.23)	0.149
Initial AFB culture test (+)	0.80 (0.44–1.47)	0.469
Initial NAAT (+)	0.54 (0.30–0.99)	0.048

*OR* odds ratio, *CI* confidence interval, *BMI* body mass index, *AFB* acid-fast bacillus, *NAAT* nucleic acid amplification test

**Fig. 2:**
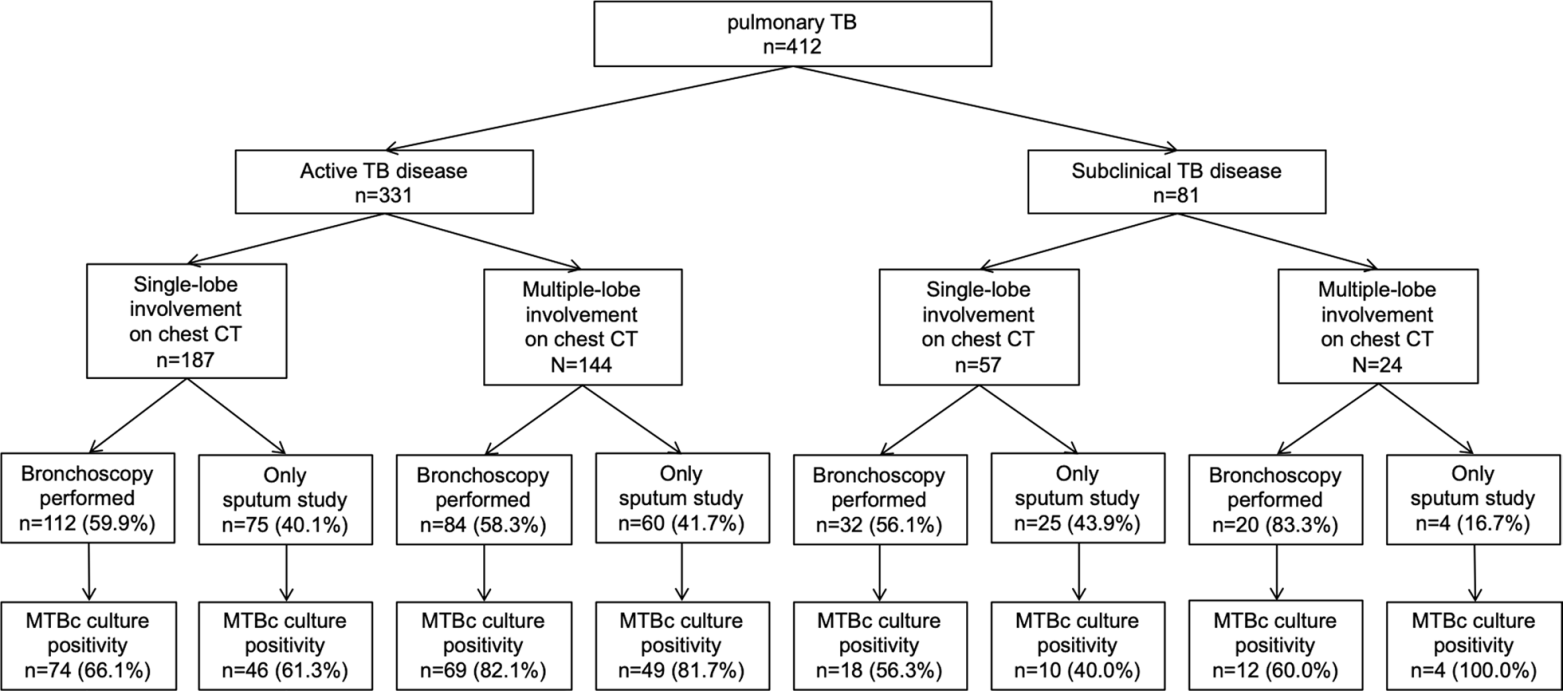
Mycobacterium tuberculosis culture results stratified by number of lobe involvement on chest computed tomography and additional performance of bronchoscopy. *TB* tuberculosis, *CT* computed tomography, *MTBc Mycobacterium tuberculosis*

We also compared the radiographic findings of chest computed tomography (CT) between subclinical and active disease patients (Table 4). Among 420 enrolled patients, 412 (98.1%) had undergone chest CT. Those with the active disease had a significantly higher proportion of multiple lobe involvement than those with subclinical disease (43.5% vs 29.6%, *p* = 0.023). Active disease was associated with radiographic findings such as consolidation (61.6% vs. 46.9%, *p* = 0.016) and fibrotic scar (19.6% vs. 9.9%, *p* = 0.039). Among 412 patients with chest CT, 248 (60.1%) had undergone bronchoscopy for microbiological tests (Fig. 2). Among 168 patients with multiple lobe involvement on chest CT, patients with subclinical disease underwent significantly more bronchoscopy than did patients with active disease (20 [83.3%] vs. 84 [58.3%], *p* = 0.020). However, the positivity of AFB culture tests between bronchoscopic and sputum specimens was similar among all patients, regardless of symptoms and extent of lobe involvement on chest CT.

**Table 4:** Comparison of chest computed tomography findings of active and subclinical tuberculosis diseases

Radiographic findings	All patients (n = 412)	Active TB disease (n = 331)	Subclinical TB disease (n = 81)
Multiple lobe involvement	168 (36.1–45.6%)	144 (38.3–48.9%)	24 (20.8–40.3%)
Tree-in-bud sign	247 (55.1–64.6%)	191 (52.3–62.9%)	56 (58.4–78.1%)
Cavitation	165 (35.4–44.9%)	129 (33.9–44.3%)	36 (34.1–55.3%)
Consolidation	242 (53.9–63.4%)	204 (56.3–66.7%)	38 (36.4–57.7%)
Fibrotic scar	73 (14.3–21.7%)	65 (15.7–24.3%)	8 (5.1–18.3%)
Atelectasis	71 (13.9–21.2%)	62 (14.9–23.3%)	9 (6.0–19.8%)
Emphysema	58 (11.1–17.8%)	45 (10.3–17.7%)	13 (9.6–25.5%)
Bronchiectasis	82 (16.3–24.0%)	67 (16.3–24.9%)	15 (11.6–28.3%)

Values were expressed as numbers with 95% confidence intervals of their proportion

*TB* tuberculosis

In the drug-susceptible cohort, comprising 75 patients with subclinical disease and 308 patients with active disease, there were 319 (83.3%) treatment-success cases, 27 (7.0%) deaths, 4 (1.0%) loss-to-follow-up cases, and 33 (8.6%) still-on-treatment cases. Among 319 patients treated successfully within 1 year, six (1.9%) recurrent cases were identified during post-treatment follow-up, and all were patients with active disease. Patients with active disease had a higher proportion of mortality during or before anti-TB treatment. Patients with subclinical disease had a higher proportion of treatment success and favourable outcome; however, the odds ratio of each treatment outcome was insignificant (Table 5). Further analysis of the association between subclinical disease and treatment outcomes among all enrolled patients, including patients with isoniazid- and rifampin-resistant TB revealed that odds ratios for mortality and treatment success were insignificant (Table 6).

**Table 5:** Comparison of treatment outcome of active and subclinical tuberculosis diseases among the drug-susceptible cohort

	Active TB disease (n = 308)	Subclinical TB disease (n = 75)	*p* value
Mortality^a^			
Number (%)	26 (8.4%)	1 (1.3%)	0.031^e^
OR (95% CI)	Reference	0.15 (0.02–1.10)	0.054
Adjusted OR^d^ (95% CI)	Reference	0.21 (0.03–1.61)	0.123
Treatment success^b^			
Number (%)	252 (81.8%)	67 (89.3%)	0.118^e^
OR (95% CI)	Reference	0.54 (0.24–1.18)	0.122
Adjusted OR^d^ (95% CI)	Reference	0.63 (0.28–1.41)	0.259
Favourable outcome^c^			
Number (%)	246 (79.9%)	67 (89.3%)	0.057^e^
OR (95% CI)	Reference	0.47 (0.21–1.04)	0.062
Adjusted OR^d^ (95% CI)	Reference	0.53 (0.24–1.18)	0.122

*TB* tuberculosis, *OR* odds ratio, *CI* confidence interval

^a^Incidence of mortality during or before anti-TB treatment

^b^Sum of cured and treatment completed cases within 1 year of anti-TB treatment

^c^Sum of treatment success and no recurrence

^d^Adjusted by age and gender

^e^Chi-square test

**Table 6:** Comparison of treatment outcome of active and subclinical tuberculosis diseases among all 420 enrolled participants

	Active TB disease (n = 339)	Subclinical TB disease (n = 81)	*p* value
Mortality^a^			
Number (%)	26 (8.3%)	1 (1.2%)	0.025^d^
OR (95% CI)	Reference	0.14 (0.02–1.04)	0.054
Adjusted OR^c^ (95% CI)	Reference	0.20 (0.03–1.55)	0.123
Treatment success^b^			
Number (%)	278 (82.0%)	71 (87.7%)	0.223^d^
OR (95% CI)	Reference	0.64 (0.31–1.32)	0.226
Adjusted OR^c^ (95% CI)	Reference	0.74 (0.36–1.53)	0.412

*TB* tuberculosis, *OR* odds ratio, *CI* confidence interval

^a^Incidence of mortality during or before anti-TB treatment

^b^Sum of cured and treatment completed cases; For tuberculosis susceptible to both isoniazid and rifampicin, treatment success was determined within 1 year of anti-TB treatment. For tuberculosis resistant to either isoniazid or rifampicin, treatment success was determined during the treatment period regardless of duration

^c^Adjusted by age and gender

^d^Chi-square test

## Discussion

This was one of the first and largest studies to evaluate the clinical characteristics of subclinical TB in an intermediate TB burden country with a low prevalence of human immunodeficiency virus (HIV) infection [14]. Bajema et al. [15] conducted a prospective study enrolling HIV-infected adults in South Africa and found that the prevalence of subclinical TB disease was common, accounting for 23% of all TB cases, and its mortality rate was similar with patients without TB. In our cohort, the prevalence of subclinical TB was 19.2%. Age < 65 years was significantly associated with subclinical disease among demographic and past medical profiles. We initially hypothesized that subclinical TB would have better treatment outcomes than active TB because of its mild nature. In our study, patients with subclinical disease had a significantly lower proportion of acid-fast bacilli smear and culture positivity and multiple lobe involvement on chest CT and lower levels of inflammatory markers compared to patients with active disease. In addition, the proportions of treatment success and favourable outcomes among the drug-susceptible cohort were higher among patients with subclinical disease; however, the difference was not statistically significant. Thus, our results revealed that although subclinical TB had a milder clinical presentation, treatment outcome was not significantly different from active TB.

The prevalence of subclinical TB varies widely across epidemiological settings, populations, and screening tools used. For example, its prevalence is generally high in active case finding studies among high-risk groups, during which all participants are screened with high-sensitivity tests [3]. According to a review of 12 national prevalence surveys in Asia between 1990 and 2012, the proportion of cases that did not report TB symptoms and were only detected due to chest X-ray screening ranged from 40% in Pakistan to 79% in Myanmar [16]. In South Korea that achieved universal health coverage in 1989, chest X-ray is a simple, inexpensive, and important health examination tool in various settings [6, 17]. For example, TB screening using chest radiography is included in the health examination performed every 1–2 years for health insurance subscribers over 40 years old. Pre-employment medical examination at many workplaces includes chest radiography. Patients who visit hospitals for other diseases or are slated for surgery undergo chest radiography. Easy access to chest radiography may have contributed to prompt detection of subclinical TB in our study, which should be emphasized when planning public health interventions for TB control because early identification of subclinical disease may reduce its transmission.

Our study showed that age < 65 years was a significant factor among demographic and clinical variables associated with subclinical TB disease. The causal relationship between age and subclinical disease cannot be confirmed; however, it might be ascribed to easy and frequent access to chest radiography among young adults, which increased detection rates of subclinical disease in South Korea. Because the proportion of elderly patients with TB is increasing in South Korea [18], early detection of subclinical disease in the elderly population is crucial to meet the WHO’s End TB target. Therefore, a pilot, TB screening project, targeting the elderly population aged ≥ 65 years was conducted in 2017 [19]. The second national TB control plan 2018–2022 designated the elderly as a high-risk group to strengthen and improve comprehensive patient management. The proportion of low body mass index in patients with the active disease was not significant but higher than that in those with the subclinical disease in our study. Because active TB is well-known to be associated with physical deconditioning, the extent to which subclinical TB affects it should be further investigated.

Current microbiological tests to diagnose active TB, such as AFB smear and culture tests and NAAT, are also employed to detect subclinical TB. The positive rate was three times higher for the NAAT than for the AFB smear test in subclinical TB, compared to two times higher in active TB. The usefulness of NAAT in subclinical TB as a point-of-care test needs to be highlighted. Moreover, the use of bronchoscopy may improve yields of microbiologic tests in patients with subclinical disease. One retrospective Korean study showed that the proportion of patients diagnosed using bronchoscopic specimens increased from 6.6% in 2005 to 26.7% in 2013 [20]. In addition, chest CT, which is widely used in routine clinical settings in South Korea, is a useful and non-invasive tool to identify subtle nodular lesions and determine disease activity to detect subclinical disease. In our study, 98% of enrolled TB patients underwent chest CT, and 83% of asymptomatic patients with multiple lobe involvement on chest CT underwent bronchoscopy. Unless other diagnostic tools are available, it is important to develop a cost-effective algorithm to diagnose subclinical disease using NAAT, chest CT, and bronchoscopy. The WHO has prioritized the development of novel tests using non-sputum-based specimens types and urine-based tests were recently developed and introduced, which may be useful in clinical point-of-care settings to diagnose TB in people living with human immunodeficiency viruses [21].

The degree of AFB smear positivity is considered an important marker for potential transmission. In our study, the rate of positivity of the initial AFB smear test in subclinical disease was only 13.6%, suggesting that these patients may pose a low risk for transmission; however, the overall contribution of subclinical disease to transmission is not yet well understood. A recent review suggested that subclinical disease might progress to an unstable state with infection taking a waxing-waning course during which precipitating factors may trigger periods of progression [4]. Therefore, a transition from smear-negative to smear-positive disease may occur depending on the host’s immunity during heterogeneous periods of subclinical disease. In a previous large cohort study, patients with smear-negative, culture-positive TB were responsible for 13% of TB transmissions [22]. Thus, we cannot confirm that subclinical disease is less infectious than active disease. A prevention strategy concerning transmission from patients with subclinical disease should also be highlighted.

This study has some limitations. First, adequate power to detect differences between treatment success and favourable outcomes in the drug-susceptible cohort was limited by the sample size. Second, the study was conducted in university-affiliated hospitals that actively participate in the public–private mix project, and more severe TB patients, who were referred from primary healthcare facilities, might have been enrolled in our study. Thus, our results cannot be inferred to other TB clinics, such as public health centres and other private hospitals. Third, we used a convenience sampling strategy to enroll study participants. Because of its non-probabilistic nature, the study lacks generalisability, which might lead to selection bias.

## Conclusions

Nearly one-fifth of adult patients with pulmonary TB were subclinical in our prospective cohort conducted in a low HIV-prevalent setting. Although subclinical TB had a milder clinical presentation and lower inflammatory markers level, its treatment outcomes were not significantly different from those of active TB. In clinical practice, patients with chest radiography suggesting TB disease without symptoms should be referred to the pulmonologist and thoroughly investigated for diagnosis and treatment. Easy and frequent access to chest radiography under the universal health coverage in South Korea might have improved prompt detection of subclinical disease, which is an important and potential target for preventing TB transmission. More researches are necessary to develop diagnostic algorithms with higher sensitivity based on currently available tools and to customize treatment strategies based on disease extent for subclinical TB.

## Data Availability

The ownership of the primary datasets lies with the Korea Centers for Disease Control and Prevention (KCDC). The datasets generated and/or analysed during the current study are available from the corresponding author on reasonable request with permission of the KCDC. The corresponding author should initially be contacted for the request accessing the raw data.

## Abbreviations

WHO: World Health Organization
TB: Tuberculosis
COSMOTB: Cohort study of pulmonary tuberculosis
AFB: Acid-fast bacilli
NAAT: Nucleic acid amplification test
CT: Computed tomography
